# Trauma-Associated Perforation of a Duodenal Ulcer With Concomitant Acute Appendicitis Presenting As Generalized Peritonitis: A Case Report

**DOI:** 10.7759/cureus.111123

**Published:** 2026-06-18

**Authors:** Hugo Eduardo Mora Moreno, Marisela Silva García, Omar J Madrigal Flores, Cesar A Duran Ramirez, Miguel A Carrillo Godinez

**Affiliations:** 1 General Surgery, Hospital General "Dr. Miguel Silva", Morelia, MEX

**Keywords:** acute appendicitis, blunt abdominal trauma, generalized peritonitis, perforated duodenal ulcer, primary duodenal repair

## Abstract

A perforated duodenal ulcer is a potentially life-threatening surgical emergency that may present with generalized peritonitis and require prompt operative management. Although most cases are related to peptic ulcer disease, clinical presentation following blunt abdominal trauma can create diagnostic uncertainty, particularly when imaging findings are non-specific or suggest an alternative intra-abdominal source. We report the case of a 68-year-old man with no significant medical history who presented with progressive abdominal pain four days after a ground-level fall. Physical examination revealed generalized peritoneal irritation, while contrast-enhanced computed tomography demonstrated large-volume free intraperitoneal fluid, diffuse bowel distension, and findings suspicious for acute appendicitis, without clear preoperative localization of the gastrointestinal perforation site. Emergent exploratory laparotomy revealed approximately 2 L of gastroduodenal contents, extensive fibrinous exudate, dense interloop adhesions, and a 1 cm perforation on the anterior wall of the first portion of the duodenum. The appendix was also enlarged, erythematous, and edematous. Biopsies of the ulcer margins were obtained, followed by primary duodenal repair, appendectomy, extensive peritoneal lavage, and drain placement. The postoperative course was uncomplicated, with gradual diet advancement and discharge on postoperative day 7. Histopathology showed perforated duodenal tissue with ischemic necrosis and edema, without dysplasia or malignancy, and confirmed acute appendicitis with mucosal ulceration and hemorrhagic changes. This case highlights that, in patients with generalized peritonitis, the decision for urgent surgical exploration should be driven primarily by clinical findings and the need for timely source control, while preoperative imaging may assist diagnosis but may not always precisely localize the perforation site.

## Introduction

Perforated peptic ulcer disease remains a life-threatening surgical emergency associated with substantial morbidity and mortality, particularly when diagnosis or operative management is delayed [1]. Duodenal ulcer perforation may rapidly progress to diffuse peritoneal contamination, generalized peritonitis, sepsis, and postoperative complications, making early recognition and timely surgical source control essential [2]. Although most perforated duodenal ulcers are related to peptic ulcer disease, non-steroidal anti-inflammatory drug exposure, or Helicobacter pylori infection, the onset of symptoms after blunt abdominal trauma may create diagnostic uncertainty. In such cases, the temporal relationship should be interpreted cautiously, as trauma may unmask symptoms from a pre-existing ulcer rather than directly cause the perforation.

Traumatic duodenal injuries are rare and should be distinguished according to mechanism, as adult duodenal trauma is more commonly associated with penetrating injury, whereas clinically significant duodenal perforation after blunt trauma is exceedingly uncommon. Blunt duodenal trauma more often presents as an intramural hematoma, particularly in the absence of major associated injuries. Therefore, in the present case, the ground-level fall should not be interpreted as definitive evidence of a primary traumatic duodenal perforation but rather as a temporally associated event that contributed to diagnostic uncertainty and may have unmasked symptoms from a pre-existing duodenal ulcer [3].

In patients presenting with generalized peritonitis, operative exploration remains central when imaging findings and physical examination suggest an acute surgical abdomen. The management of complicated intra-abdominal infections relies on early diagnosis, adequate source control, appropriate antimicrobial therapy, and physiologic support [4]. Similarly, contemporary surgical infection guidelines emphasize that delayed or incomplete source control is associated with worse outcomes in patients with intra-abdominal sepsis [5].

Acute appendicitis remains one of the most common causes of acute abdominal pain in adults, and diagnosis usually depends on the integration of clinical findings, laboratory evaluation, and imaging [6]. However, in the setting of diffuse peritonitis from another intra-abdominal source, appendiceal inflammation may represent either a concomitant pathology or a confounding radiologic and intraoperative finding. We present the case of an older adult who developed generalized peritonitis after blunt abdominal trauma and was found to have a perforated duodenal ulcer with concomitant acute appendicitis, successfully managed with primary duodenal repair, appendectomy, and peritoneal lavage.

## Case presentation

A 68-year-old man with no significant medical history and no regular medication use presented to the emergency department with progressively worsening abdominal pain that began after a ground-level fall four days before admission. The fall was a low-energy mechanism, and no other associated traumatic injuries were identified during the initial evaluation. The patient had no abdominal wall ecchymosis, abrasions, external signs of trauma, or evidence of major associated injury. Because this mechanism would be unusual for a primary traumatic duodenal perforation, the fall was interpreted as a temporally associated event that contributed to diagnostic uncertainty rather than definitive evidence of direct traumatic duodenal injury. The pain began shortly after the fall and was initially localized to the epigastrium. He described it as severe, sharp, and stabbing, with gradual progression in intensity over the following days. His symptoms were associated with nausea, progressive abdominal distension, constipation, and difficulty walking because of pain. Due to persistent clinical deterioration, he sought medical attention.

On admission, the patient was alert, oriented, and mildly dehydrated. He was hemodynamically stable. Abdominal examination revealed marked distension, diffuse tympany, hypoactive bowel sounds, and generalized tenderness. Involuntary guarding and rebound tenderness were present, consistent with generalized peritoneal irritation. No abdominal wall hernias, external signs of trauma, or previous surgical scars were identified.

Initial laboratory findings are summarized in Table 1. Despite the severity of the intra-abdominal findings, laboratory evaluation showed no significant leukocytosis or marked inflammatory response.

**Table 1:** Initial laboratory findings on admission.

Laboratory test	Result	Reference range
White blood cell count	8.3 × 10³/µL	4.5-10 × 10³/µL
Hemoglobin	15.8 g/dL	13-17 g/dL
Platelet count	223 × 10³/µL	150-400 × 10³/µL
International normalized ratio	1.34	0.8-1.3
Blood glucose	72 mg/dL	74-109 mg/dL
Serum creatinine	1.49 mg/dL	0.6-1.2 mg/dL
Aspartate aminotransferase	30 U/L	0-40 U/L
Alanine aminotransferase	9 U/L	0-41 U/L

Contrast-enhanced computed tomography (CT) of the abdomen showed large-volume, free intraperitoneal fluid surrounding the liver and extending into the right paracolic gutter, associated with diffuse bowel distension and extensive inflammatory changes (Figure 1). The appendix measured approximately 8 mm in diameter and was associated with periappendiceal inflammatory changes, raising concern for acute appendicitis (Figures 2, 3). No definitive pneumoperitoneum or clear site of gastrointestinal perforation was identified preoperatively.

**Figure 1:**
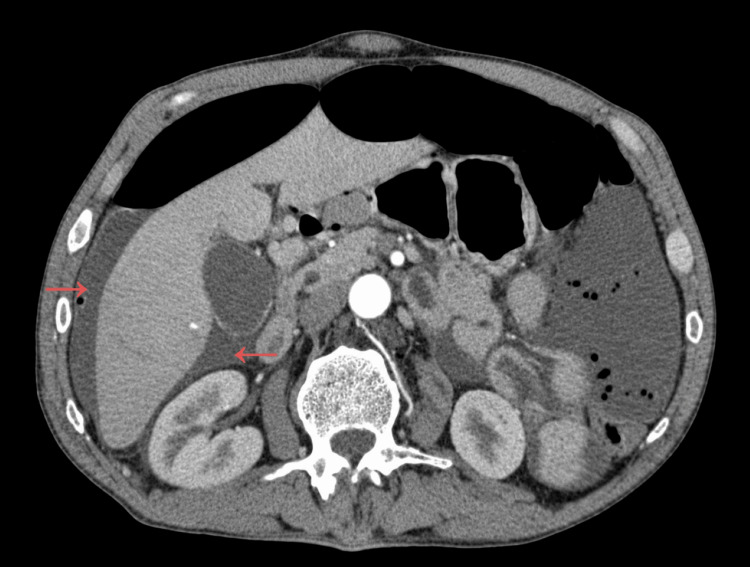
Axial contrast-enhanced abdominal computed tomography (CT) scan showing free intraperitoneal fluid. Red arrows indicate free intraperitoneal fluid surrounding the liver and extending into the right paracolic gutter, associated with diffuse bowel distension and extensive intra-abdominal inflammatory changes.

**Figure 2:**
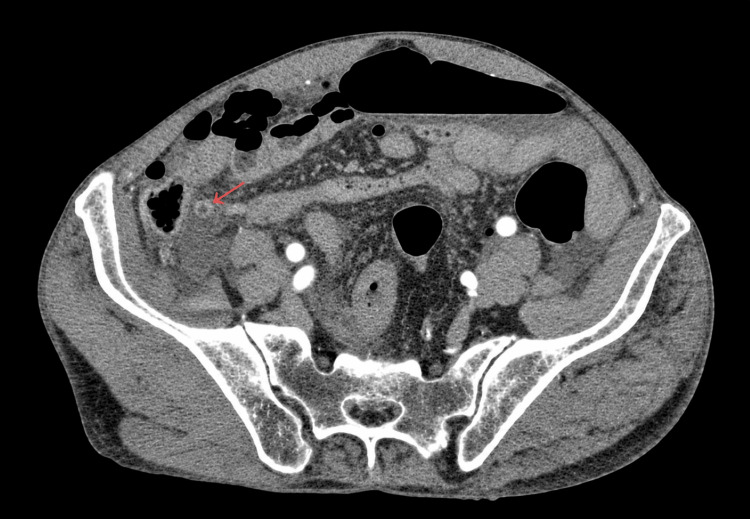
Axial CT findings suspicious for acute appendicitis. The red arrow highlights the enlarged appendix, measuring approximately 8 mm in diameter. CT: computed tomography.

**Figure 3:**
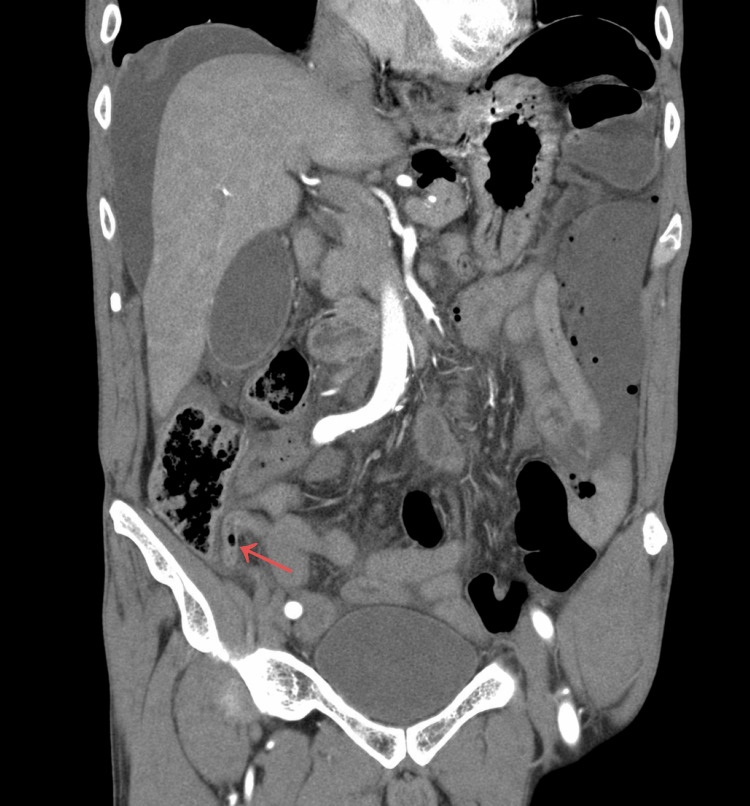
Coronal CT reconstruction showing appendiceal inflammatory changes. The red arrow highlights the enlarged appendix in the right lower quadrant, with surrounding fat stranding and localized inflammatory changes. CT: computed tomography.

Given the clinical findings of generalized peritonitis and CT evidence of an acute surgical abdomen, the patient underwent emergent exploratory laparotomy. Upon entry into the peritoneal cavity, approximately 2 L of gastroduodenal contents were encountered, along with extensive fibrinous exudate and dense interloop adhesions (Figure 4). Careful exploration revealed a 1 cm perforation on the anterior wall of the first portion of the duodenum, with active leakage of gastric contents into the peritoneal cavity (Figure 5). The appendix was also erythematous, edematous, and enlarged, consistent with acute appendicitis (Figure 6).

**Figure 4:**
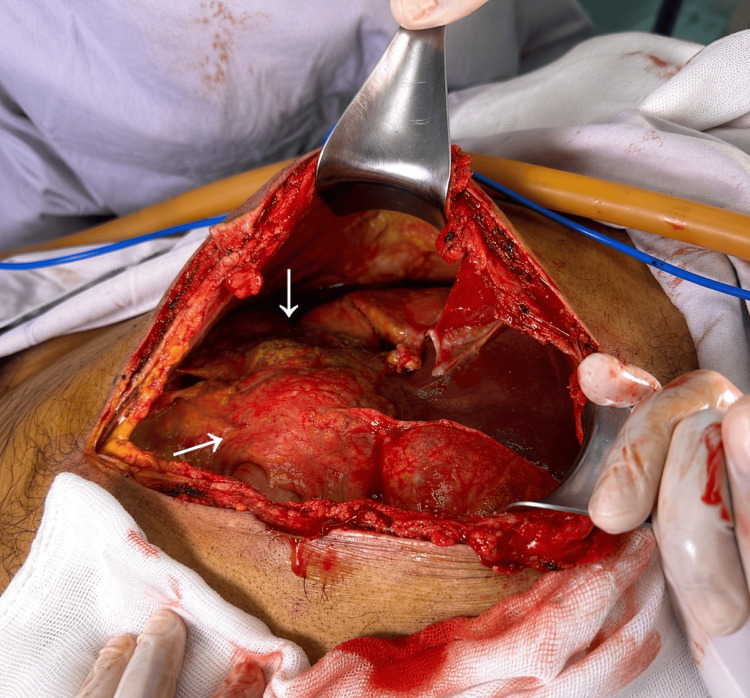
Exploratory laparotomy demonstrating generalized peritonitis. The white arrows highlight fibrinous deposits and dense inflammatory adhesions between adjacent bowel loops, consistent with an advanced intra-abdominal inflammatory process.

**Figure 5:**
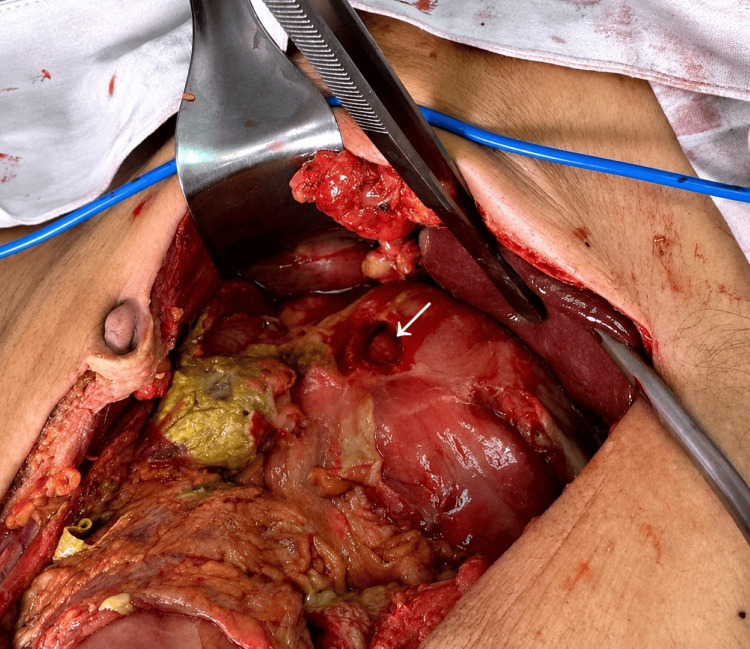
Perforated duodenal ulcer identified during surgical exploration. The white arrow indicates the perforation on the anterior wall of the first portion of the duodenum, surrounded by inflammatory changes and gastroduodenal contamination.

**Figure 6:**
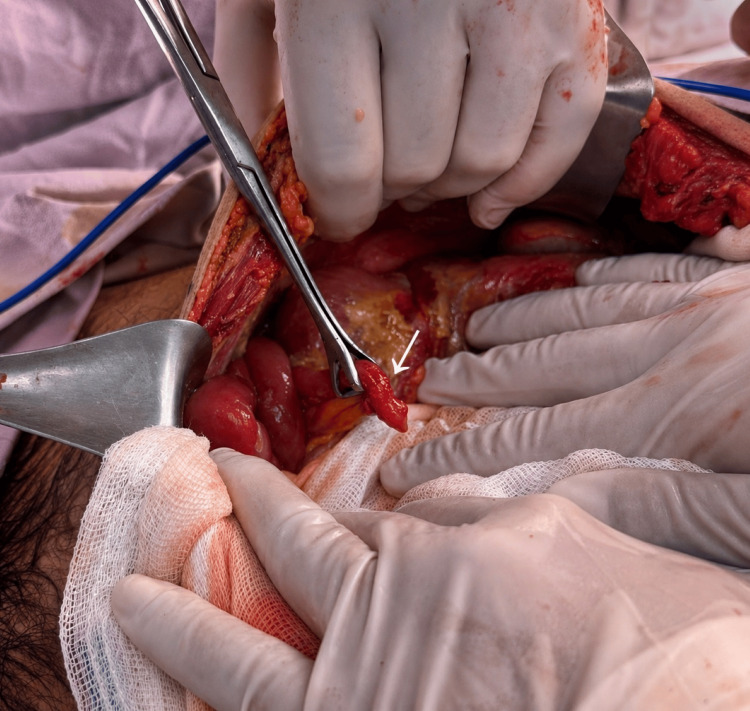
Intraoperative findings of acute appendicitis. The white arrow highlights the inflamed appendix, which appears hyperemic and enlarged, with surrounding inflammatory tissue and fibrinous exudate.

Biopsy samples were obtained from the margins of the duodenal ulcer to exclude underlying malignancy. The duodenal perforation was repaired primarily using absorbable 2-0 chromic catgut sutures with a Connell-Mayo closure, reinforced with interrupted 2-0 silk Lembert sutures. Extensive peritoneal lavage was then performed. Because of the gross inflammatory appearance of the appendix and the risk of future appendiceal complications, appendectomy was performed during the same operation. The appendiceal stump was managed using a modified purse-string technique with a 2-0 Vicryl suture. A nasogastric tube and passive intra-abdominal drains were placed before abdominal wall closure.

The patient was admitted to the general surgery ward for postoperative monitoring. Broad-spectrum antibiotic therapy with ceftriaxone and metronidazole was initiated, together with intravenous fluid resuscitation, analgesia, gastric decompression through the nasogastric tube, and bowel rest during the first three postoperative days.

His postoperative course was favorable. Drain output remained serous, with no evidence of enteric or biliary leakage, and nasogastric drainage was minimal. Laboratory evaluation 72 hours after surgery showed no leukocytosis or biochemical evidence of persistent inflammatory response. On postoperative day 4, oral liquid intake was initiated after clamping the nasogastric tube. A bedside oral dye challenge using a colored liquid showed no evidence of leakage through the abdominal drains, and the patient tolerated oral intake without clinical deterioration. On postoperative day 5, his diet was advanced to solid food without abdominal pain, nausea, vomiting, or recurrent distension, allowing removal of the nasogastric tube. He subsequently passed flatus and bowel movements, confirming recovery of gastrointestinal function.

By postoperative day 7, the abdominal drains were removed. The patient remained afebrile, hemodynamically stable, and asymptomatic, with adequate bowel function. Follow-up laboratory studies showed no evidence of ongoing inflammatory response or intra-abdominal infection, and he was discharged in stable condition.

At the two-week outpatient follow-up, the patient remained asymptomatic, with adequate oral tolerance and normal bowel habits. Histopathological examination of the duodenal ulcer margins showed duodenal tissue with perforation, ischemic necrosis, and edema, without dysplasia or malignancy. Histopathological examination of the appendix revealed acute appendicitis with mucosal ulceration and extensive hemorrhagic changes. Given the favorable postoperative recovery and absence of malignant findings, the patient was discharged from further surgical follow-up.

## Discussion

This case highlights the importance of prioritizing clinical findings and timely source control in patients with generalized peritonitis. In our patient, physical examination demonstrated generalized peritoneal irritation, and CT showed large-volume free intraperitoneal fluid, diffuse pneumoperitoneum, bowel distension, and inflammatory changes. Although these CT findings established the presence of an acute surgical abdomen, the precise site of gastrointestinal perforation was not clearly localized preoperatively, which may occur in gastroduodenal perforation when direct findings are obscured by diffuse contamination [7]. The concomitant finding of an enlarged appendix with periappendiceal inflammatory changes suggested acute appendicitis and contributed to uncertainty regarding the primary source of contamination; however, appendiceal imaging findings may be difficult to interpret in patients with equivocal or overlapping CT findings [8]. Importantly, this uncertainty did not alter the indication for operative management.

Emergent exploratory laparotomy was indicated because the patient had an acute abdomen with generalized peritonitis, supported by CT findings of free intraperitoneal fluid and pneumoperitoneum. In this setting, surgical management should prioritize timely source control, evacuation of contaminated fluid, repair of the gastrointestinal defect, and adequate peritoneal lavage [9]. Although laparoscopic repair may be appropriate in selected patients with perforated peptic ulcer disease, an open approach remains justified when there is extensive contamination, dense adhesions, diagnostic uncertainty, or the need for full abdominal exploration [10]. In our case, the presence of approximately 2 L of gastroduodenal contents, extensive fibrinous exudate, dense interloop adhesions, and a concomitantly inflamed appendix supported the use of open exploration.

The 1 cm perforation identified on the anterior wall of the first portion of the duodenum was managed with primary repair and reinforced with interrupted seromuscular sutures. Primary repair remains a widely used technique for perforated peptic ulcers, particularly when the defect is small, the surrounding tissue is suitable for closure, and adequate source control can be achieved [11]. The optimal repair technique for perforated peptic ulcers continues to be debated, especially regarding primary closure alone versus reinforcement with an omental patch or other adjunctive techniques [12]. In this patient, the decision to perform a reinforced primary closure was supported by the small size and anterior location of the perforation, the absence of gross malignant features, and the feasibility of achieving secure closure after biopsy of the ulcer margins [13].

Because the patient’s epigastric pain began after a low-energy ground-level fall, trauma was initially considered in the differential diagnosis; however, perforation of the adult duodenum from blunt trauma is exceedingly rare, particularly in the absence of other major associated injuries. Therefore, this relationship should be interpreted cautiously, as the fall may have contributed to diagnostic uncertainty or unmasked symptoms from a pre-existing duodenal ulcer rather than representing definitive evidence of a primary traumatic duodenal perforation. Contemporary trauma literature emphasizes that blunt duodenal injuries are uncommon, may be difficult to diagnose early, and require individualized operative management based on patient physiology, injury severity, tissue viability, contamination, and associated injuries [14]. In our patient, the operative finding was more consistent with a perforated duodenal ulcer than a destructive traumatic duodenal injury, making primary repair reasonable in the absence of extensive tissue loss or devascularization [15]. The favorable outcome without a duodenal leak also supports the appropriateness of a less complex repair in this specific setting, although more complex repairs may be required in higher-grade traumatic duodenal injuries or unstable patients [16].

Postoperative management focused on early recognition of leakage, infection, and delayed gastrointestinal recovery. In this case, clinical monitoring, drain characteristics, nasogastric output, serial laboratory evaluation, and a bedside oral dye challenge were used to assess the integrity of the repair before diet advancement. Routine postoperative contrast imaging after perforated peptic ulcer repair has not consistently shown improved detection of clinically significant leaks and may prolong hospitalization when used indiscriminately [17]. The use of intra-abdominal drains after peptic ulcer perforation repair also remains controversial; although some recent evidence suggests that omitting drains may be safe in selected patients, drains can still provide useful clinical information in cases with severe contamination, delayed presentation, or concern for postoperative leakage [18]. Given the extensive contamination encountered intraoperatively in our patient, drain placement was a reasonable adjunct to postoperative surveillance.

This case also underscores the importance of carefully evaluating concomitant intra-abdominal findings during surgical exploration. The appendix was enlarged, erythematous, and edematous, and histopathology demonstrated acute appendicitis with mucosal ulceration and extensive hemorrhagic changes. However, based on the available findings, it is not possible to determine whether the appendiceal inflammation represented a synchronous primary appendicitis or secondary inflammatory involvement in the setting of generalized peritonitis. Therefore, appendectomy during the same procedure was considered reasonable because of the gross intraoperative appearance of the appendix and the potential for persistent diagnostic uncertainty if an abnormal appendix had been left in place. Despite the delayed presentation and extensive peritoneal contamination, the patient had an uncomplicated recovery, tolerated diet advancement, had no clinical evidence of leakage, and was discharged in stable condition by postoperative day 7. This favorable course is clinically relevant because surgery for perforated peptic ulcer continues to be associated with substantial short-term morbidity and mortality, particularly in older patients and those presenting with physiologic compromise [19]. A limitation of this report is the short follow-up period of two weeks, which restricts assessment of late complications, ulcer recurrence, and long-term gastrointestinal outcomes. Further outpatient evaluation, including assessment for *Helicobacter pylori* infection and consideration of endoscopic follow-up, would be appropriate to reduce the risk of recurrent peptic ulcer disease.

## Conclusions

This case emphasizes the importance of prompt operative management in patients with an acute abdomen and generalized peritonitis. In this patient, physical examination findings and CT evidence of free intraperitoneal fluid and pneumoperitoneum supported the need for urgent surgical exploration and source control. Exploratory laparotomy revealed a perforated duodenal ulcer with an abnormal, inflamed appendix, both of which were managed during the same operation. Primary repair of the duodenal perforation, appendectomy, extensive peritoneal lavage, and careful postoperative monitoring resulted in an uncomplicated recovery. Although the onset of symptoms after a low-energy ground-level fall contributed to diagnostic uncertainty, this temporal relationship should be interpreted cautiously and should not be considered definitive evidence of primary traumatic duodenal perforation.
